# The late preterm in low income

**DOI:** 10.1186/1824-7288-40-S2-A28

**Published:** 2014-10-09

**Authors:** PE Villani, G Vellani, GP Chiaffoni, R Magaldi, E Padovani, A Ricchini, P Stillo, B Tomasini, F Uxa, D Trevisanuto, M Usuelli

**Affiliations:** 1 SC Neonatologia e Terapia Intensiva Neonatale, Dipartimento Materno-Infantile, AO “C.Poma”, Mantova, Italy; 2 Neonatologia, Dipartimento Integrato Materno-Infantile, AO Universitaria, Policlinico Modena, Italy; 3 SC Pediatria e Neonatologia, ULSS 7 Conegliano e Vittorio Veneto, Italy; 4 SC Neonatologia e Terapia Intensiva Neonatale, Ospedali Riuniti AOU Foggia, Italy; 5 SC Patologia e Terapia Intensiva Neonatale ,Azienda Ospedaliera e Universitaria Integrata, Verona, Italy; 6 UO Neonatologia e Terapia Intensiva Neonatale, Ospedale dei Bambini, Brescia, Italy; 7 SC Chirurgia Pediatrica, Azienda Ospedaliera Universitaria Meyer, Firenze, Italy; 8 UO di Terapia Intensiva Neonatale, Azienda Ospedaliera Universitaria “Le Scotte”, Siena, Italy; 9 IRCCS Materno Infantile, Azienda Burlo Garofolo, Trieste, Italy; 10 Dipartmento di Salute della Donna e del Bambino, Scuola di Medicina, Università di Padova, AO Padova, Italy; 11 NICU, Fond. IRCCS Cà Granda Osp. Maggiore Policlinico Milano, Italy

## 

Millennium Development Goals (MDGs) represent the widest commitment in history to addressing global poverty and health. MDG-4 commits international community to reducing mortality in under 5 by two-thirds between 1990 and 2015. According to Lancet Neonatal Survival Series (LNSS), since 2000, child mortality after the first month (from 29 days to 5 years) fell by a third [1]. Meanwhile, little improvement has been achieved toward reduction of neonatal mortality (NMR), resulting in neonatal deaths (ND) constituting an increasing proportion of all under-5 deaths. Estimates from LNSS show that 38% of all deaths in children younger than age 5 years occur in the neonatal period, 99% in 39 low-income countries where the average NMR is 33/1,000 [2], 50% occur at community level and 50% at hospital level. The members of SIN study group “neonatal care in developing countries (PVS)” experience cooperation with several PVS maternity hospitals. Preterm birth is a major health issue and most important clinical problem, especially in PVS [3]. In 2010, preterm birth from 184 countries amount to 14.9 million, representing 11.1% of livebirths [4] with geographical variations ranging from 5% in European countries up to 18% in African countries. About 75% of preterm births are late preterm. Compared with term infants, late preterm have higher risk of mortality, morbidities including hypothermia, hypocalcemia, hyperbilirubinemia, sepsis, seizures, respiratory distress, feeding difficulty, readmission and neurodevelopmental problems [5-8]. Neonatal setting in PVS deeply differs from western: WHO consider newborns <28 weeks gestational age out of threshold of life with chances of survival, strengthening the emphasis of late preterm survival. This depends on department organization, a minimal nurse-patient ratio and defined protocols and procedures rather than intensive care setting [9]. In addition prematurity is somehow a difficult diagnosis in PVS: usually women cannot define the first day of their last menstruation period; even harder is to access ultrasound. Therefore that many senior neonatologists in PVS prefer to ignore, after birth, the presumptive gestational age and to manage newborns only according to birth weight. Standardized protocols do not differentiate procedures, for example, in 2 newborns of 1700g, 1 AGA and 1 SGA [10]. Achievement of MDG4 on childhood mortality will remain unattainable in developing countries unless interventions targeted at reducing NM are instituted; late preterm babies are the most achievable target [11]. The essential package for neonatal survival include neonatal resuscitation, hygiene, management of hypothermia, LBW, neonatal infections, hyperbilirubinaemia, kangaroo mother care and breastfeeding support, especially for small, LBW newborns [12].
